# The Eternal Entity: Fear–ICD 11 Diagnosis of Phobia Compared to 9th Century Phenomenology

**DOI:** 10.1192/bjo.2026.11185

**Published:** 2026-06-30

**Authors:** Ayan Adnan Shaukat, Usman Abdul-Quayum

**Affiliations:** 1 NHS Lanarkshire, Motherwell, United Kingdom; 2 University of Glasgow, Glasgow, United Kingdom

## Abstract

**Aims:**

This study explores the historical roots of phobic disorders through an analysis of Abu Zayd al-Balkhi’s ninth-century written venture into mental health and wellbeing, “*Sustenance of the Soul*”. It compares al-Balkhi’s descriptions, differentiation, and management of phobia with the current ICD-11 framework. Previous comparative research has demonstrated consistencies in his writings when compared to contemporary framework. As such, we hypothesised that al-Balkhi’s account would show substantial conceptual overlap with modern nosology while offering a more integrated phenomenological perspective that remains relevant to current psychiatric practice.

**Methods:**

A multi-stage content analysis was undertaken using the manifest analysis method. First, relevant thematic premises were identified through examination of al Bakhli’s text. Second, focussing on aetiology, symptomatology, and therapeutic approaches, psychological terminology and their implied meanings were extracted. Finally, these findings were compared directly with the ICD-11 phobia-related classification and associated diagnostic constructs. The analytical process and mappings were independently cross-checked by a secondary researcher.

**Results:**

Al-Balkhi described phobia as an excessive, maladaptive fear response–referred in his work as “terror”–that exceeds the individual’s control and impairs functioning. He distinguished normative fear states from the pathological using description of threat appraisal and temporal proximity, paralleling modern distinctions between general anxiety and phobia. He also described symptoms of physiological arousal and cognitive interferences during fear states, including restricted clarity of thought. Management strategies included education regarding feared stimuli and desensitisation-like techniques, anticipating the core principles of contemporary psychological treatments.

**Conclusion:**

Al-Balkhi’s account of phobia correlates to a significant degree with ICD-11 categorisations of anxiety and phobic disorders. Although some of the ideology lacks modern political correctness which reflects its era, the underlying clinical observations are notably consistent with modern phenomenology and psychologically informed care. These findings support the value of historical psychiatric scholarship in refining contemporary understanding and suggests that pre-modern consolidative models may still enrich current diagnostic and therapeutic approaches.

